# Do we need to treat asymptomatic catheter-related thrombosis?

**DOI:** 10.1016/j.rpth.2024.102450

**Published:** 2024-05-24

**Authors:** Philippe Debourdeau, Jean Philippe Hugret, Théodore Debourdeau

**Affiliations:** 1 Oncology Supportive Care Department, Hôpital Joseph Imbert, Arles cedex, France; 2 Anesthesiology Department, Hôpital Joseph Imbert, Arles cedex, France; 3 Anesthesiology Department, Hôpital Saint Eloi, Montpellier, France

To the Editor,

We read with interest the article by Cominacini et al. [1]. Two main findings emerge from this study. First, contrary to previous articles, it does not demonstrate a relationship between fibrin sheaths and venous thromboses, whether superficial or deep. This observation could be explained by the localization of the fibrin sheath at the distal end of the catheter, whereas superficial thromboses occur at the proximal level of catheter insertion. So, it may have been necessary to separate superficial and deep thromboses in the analysis. Second, the question raised by the authors in the discussion is whether asymptomatic thromboses on central catheters should be treated. Currently, there are no recommendations. However, there appears to be some confusion in the discussion as the authors cite 4 references (35-38) that pertain to superficial thromboses of the lower limbs, for which anticoagulant treatment is recommended due to their risks of extension into the deep network and of embolic migration. The results of treatment for anticoagulated asymptomatic thromboses from 3 studies included in a recent literature review [2] and 2 prospective observational studies [3,4] without anticoagulants appear to be comparable. Identifying predictive factors for symptomatic thrombosis occurrence on ultrasound and, above all, conducting a randomized study comparing anticoagulants to therapeutic abstention in asymptomatic thromboses could help answer this question.
